# Effect of sodium hypochlorite and hyaluronic acid in subgingival re-instrumentation – a randomized clinical trial

**DOI:** 10.1186/s12903-026-08694-9

**Published:** 2026-05-30

**Authors:** Gert Jungbauer, Fabienne Stalder, Steffen Müller, Anton Sculean, Sigrun Eick, Holger Jentsch

**Affiliations:** 1 Department of Periodontology, School of Dental Medicine, University of Bern, Bern, Switzerland; 2 Medical Faculty, University of Leipzig, Leipzig, Germany; 3 Department of Maxillofacial Surgery, University of Regensburg, Regensburg, Germany

**Keywords:** Sodium hypochlorite, Hyaluronic acid, Subgingival re-instrumentation, Supportive periodontal care

## Abstract

**Background:**

Adjunctive antimicrobials may improve the outcome of subgingival instrumentation, but due to the lack of evidence no antimicrobial agent is recommended for subgingival re-instrumentation (SRI). The aim of this study was to clinically, immunologically and microbiologically evaluate the potential additional effects of amino-acid sodium hypochlorite (AA-NaOCl) and cross-linked hyaluronic acid (xHA) to SRI at 3 and 6 months.

**Methods:**

At the time point of reevaluation during systematic periodontal therapy, eligible patients were allocated to either control group receiving SRI of residual pockets, or test group with the adjunctive AA-NaOCl and xHA. Per patient 2 study sites were selected and clinical periodontal parameters (PD, CAL, BOP, and pocket closure), immunological biomarkers and 8 major periopathobionts were analyzed at baseline, after 3 and 6 months. Detection of IL-1β and MMP-8 was carried out using ELISA, the abundance of the periopathobionts by qPCR.

**Results:**

Improvements in the clinical parameters were demonstrated in all 42 included patients. In the test group an additional PD reduction by 0.50 mm (1.38, 95% CI range: 1.11–1.65 vs. 0.88, 95% CI range: 0.62–1.14; *p* = 0.008) and CAL gain by 0.57 mm (1.07, 95% CI range: 0.70–1.44 vs. 0.50, 95% CI range: 0.24–0.76; *p* = 0.017) was found compared to the control group. The percentage of residual pockets decreased by 88.1% (*n* = 37) in the test, and 38.1% (*n* = 16) in the control group. There was no significant change in the immunological parameters. The abundance of 5 periopathobionts significantly decreased in the test group.

**Conclusions:**

The adjunctive application of AA-NaOCl and xHA significantly improved the clinical and microbiological outcome 3 and 6 months after SRI.

**Trial registration:**

This trial was registered in the German Clinical Trials Register (ID: DRKS00017415 at 03/06/2019).

**Supplementary Information:**

The online version contains supplementary material available at 10.1186/s12903-026-08694-9.

## Introduction

 Periodontal disease is considered the most common chronic inflammatory, non-communicable disease with a prevalence of approximately 50% for mild to severe forms [1]. The decisive treatment factor is the mechanical removal of the subgingival periopathogenic biofilm and calculus to reestablish the ecological balance in the pocket microenvironment [2]. Studies revealed a high percentage of uncleaned surface in multi-rooted teeth and in deep pockets after non-surgical debridement [3–5]. Many attempts were made to support the subgingival instrumentation (SI) with adjunctive antimicrobials. Currently, no recommendation for any of the established adjuncts exists due to the lack of scientific evidence [6], neither for physical methods, like lasers or photodynamic therapy [7], nor locally administered antimicrobials including antibiotics [8]. Regarding adjuvant antimicrobials after subgingival re-instrumentation (SRI), a systematic review failed to show differences in the clinical outcomes for any of the adjuvants [9]. Periodontal pockets with residually increased probing depths are recolonized by periopathogens within a few weeks [10]. Therefore, the application of biofilm-degrading sodium hypochlorite (NaOCl) and hyaluronic acid (HA) might be an innovative approach.

Sodium hypochlorite (2.5–5.25%) is widely used in endodontic treatment due to its strong antibacterial capacity [11], but also evidence of further indications, including periodontal and peri-implant diseases, is growing [12, 13]. A gel formulation with lower concentrations mixed with an amino-acid buffer (AA-NaOCl; Perisolv, Regedent, Dettelsbach, Germany) was developed for application in the periodontal pocket; in-vitro effects against a pre-formed periodontal multi-species biofilm and the extracellular matrix were shown [14]. The anti-biofilm activity decreased time-dependently, showing sufficient efficiency within 20 min after mixing [15].

Hyaluronic acid (HA) is a macromolecule consisting of the disaccharides D-glucuronic acid and N-acetyl-D-glucosamine or N-acetylglucosamine with different chain lengths [16]. Hyaluronan exhibits high hydrophilicity and water retention [17] and demonstrated a proliferative, migratory and wound healing effect on human gingival fibroblasts [18], periodontal ligament cells [19], and cementoblasts [20], and upregulated collagen-maturation related genes (MMP-1, TIMP-1, LOX) in vitro [21].

The “clean and seal” protocol by means of application of an AA-NaOCl gel for bacterial clearance and a cross-linked HA (xHA) gel for blood clot stabilization [22], is supposed to improve the clinical outcome of SI [23] and SRI [24]. Histologically the formation of new periodontal attachment could be demonstrated after the non-surgical application of AA-NaOCl and xHA in an animal model [25]. To our knowledge, there is no data on the change of periodontal microbiota after application of AA-NaOCl and xHA during SRI. The aim of this clinical trial was to investigate the effect of the adjunctive application AA-NaOCl and xHA on the additional reduction of probing depth, but also on further clinical, microbiological and immunological outcomes during SRI.

## Materials and methods

### Study design

This single-center, prospective randomized clinical trial (RCT) was registered in the German Clinical Trials Register (ID: DRKS00017415), conducted according to the Declaration of Helsinki and the CONSORT criteria [26] and approved by the Ethics committees of the Universities of Leipzig (427/17-ek, 23/01/2018) and Regensburg, Germany (21-2666-103, 02/11/2021). Participants diagnosed with periodontitis stage III or IV grade B or C were recruited in a private dental practice in Germany from February 2022 to December 2023. After a written informed consent was obtained, patients at the time-point of re-evaluation at least 3 months after step 2 or 3 or during step 4 (SPC) of the systematic periodontal therapy regime according to the EFP S3 clinical guidelines [6] were included. The inclusion criteria were completion of the active periodontal therapy, ≥ 16 teeth, ≥ 40 years of age, 2–8 sites with PD ≥ 5 mm, or PD ≥ 4 mm and positive for BOP for non-adjacent teeth, good oral hygiene indicated by an interproximal plaque index (API) ≤ 35%. Exclusion criteria were periodontitis associated with systemic diseases, intake of immunosuppressive or immunomodulating drugs, antibiotic therapy within the last 3 months, pregnancy or lactation, cancer, cigarette consumption (> 5 cigarettes per day), allergy to components of the used adjuncts, special dietary habits (e.g. vegan diet). At routine appointments during the periodontal treatment sequence at the above-mentioned occasions, the individuals were checked for eligibility. Participants were then randomly assigned to either test or control group (1:1; two parallel treatment arms) using a computer-generated block randomization (block size of 2). The allocation was concealed until the end of statistical analysis. The clinician was informed about the allocation at the beginning of the treatment.

### Treatment

All clinical assessments and periodontal treatments were performed by one experienced periodontist (G.J.). At all three timepoints (baseline, after 3 and 6 months) a periodontal chart was recorded including PD, CAL, and BOP. PD and CAL were measured using a 1-mm-scaled periodontal probe (PCP UNC15, HuFriedy, Frankfurt am Main, Germany). An overview over the study timeline is provided in the supplementary data (S1).

All patients were enrolled in a SPC program. The session consisted of oral health reinstruction and remotivation and a supragingival professional plaque removal using air polishing (supragingival handpiece, Airflow Handy 3.0, EMS, Nyon, Switzerland) and erythritol powder (perio plus, EMS, Nyon, Switzerland). Mineralized biofilm was removed with an air scaler (SONICflex Quick 2008 L, KaVo, Biberach, Germany), if present.

SRI was performed at all sites (between 2 and 8 sites per patient) with PD ≥ 5 mm or PD ≥ 4 mm and BOP+ (residual pockets). In every patient the two sites with the highest PD (non-adjacent, single- and multi-rooted teeth) were selected as study sites. If PD was equal in 2 or more pockets, the more distal site was chosen. The SRI was performed in local anesthesia containing articaine and epinephrine (Ubistesin 1/100.000, 3 M, Neuss, Germany) and instrumentation using Gracey curettes (mini five, HuFriedy, Frankfurt am Main, Germany), air scaler (SONICflex Quick 2008 L, KaVo, Biberach, Germany), and air polishing (Airflow Handy 3.0, EMS, Nyon, Switzerland) with erythritol powder (supragingival handpiece, perio plus, EMS, Nyon, Switzerland). To remove the debris and the remaining powder, the pockets were rinsed with 0.9% saline.

**Fig. 1:**
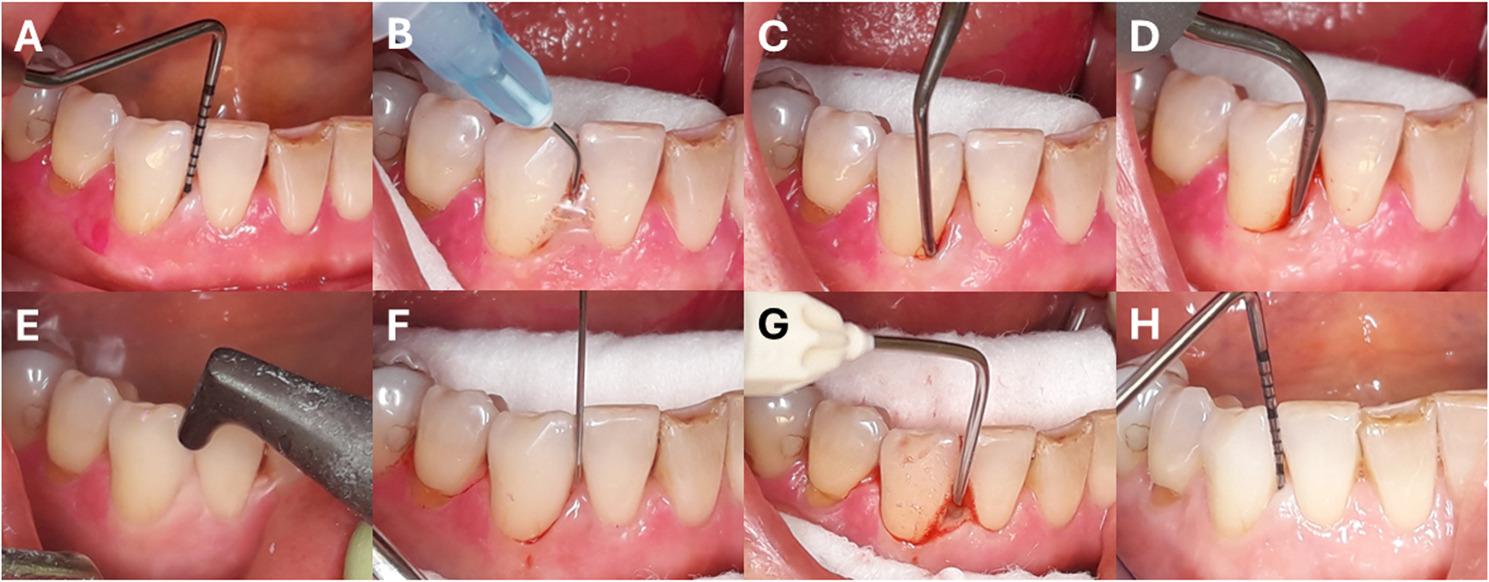
Clinical treatment protocol for the test group; PD of 4 mm and BOP+ at baseline (**A**), application of AA-NaOCl (**B**), mechanical instrumentation with Gracey curette (**C**) and airscaler (**D**), air polishing (**E**), rinsing with NaCl (**F**), and application of xHA (**G**); pocket closure 6 months after treatment (**H**), AA-NaOCl: amino-acid sodium hypochlorite, BOP: bleeding on probing, PD: probing depth, xHA: cross-linked hyaluronic acid

In the test group, additionally freshly mixed (< 20 min before application) AA-NaOCl gel (Perisolv, Regedent, Dettelbach, Germany) was instilled for 60 s prior to instrumentation, when changing from hand curettes to air scaler, and after completing the mechanical cleaning. Then, the pocket was thoroughly rinsed with 0.9% saline and xHA (hyaDent BG, Regedent, Dettelbach Germany) was applied (Fig. 1A-H). After one week, xHA was again subgingivally instilled. Three months later (T1), the persisting pockets in both groups were again retreated in the allocated therapy regime.

### Biomarker and microbiology

Samples for biomarkers and microbial diagnostics of the study sites were collected at T0, T1, and T2. The sites were isolated with cotton rolls, and the gingival margin was gently air-dried. Gingival crevicular fluid (GCF) was sampled by inserting paper strips (Periopaper, Oraflow, New York, USA) 0.5–1 mm into the gingival sulcus and kept for 60 s. Afterwards, sterile endodontic paper points (ISO 60, Coltene, Langenau, Germany) were inserted into the pocket until little resistance and left for 60 s. Paper points contaminated with blood were discarded. Paper strips were pooled and 10 µl protease inhibitor was added before being stored at -80° C until analysis. The paper points were pooled and stored at -20° C upon analysis. The samples were pseudonymized by consecutive numbers and the scientist who carried out the analysis in the laboratory was blinded to group assignment (test vs. control).

Interleukin-1beta (IL-1β) and matrix metalloproteinase-8 (MMP-8) were quantified using commercially available enzyme-linked immunosorbent assays (ELISA) kit (R&D Systems Europe Ltd., Abingdon, UK) according to the manufacturer’s instruction. The paper-strips were eluted at 4 °C in 750 µl PBS (SigmaAldrich, St. Louis, USA) overnight and centrifuged at 400 g for 2 min. After mixing the supernatant, 100 µl aliquots were used for analysis. The detection levels were 1 pg IL-1β and 100 pg MMP-8/sample.

The abundance of 8 major periopathobionts (*A*ggregatibacter *actinomycetemcomitans*, *Porphyromonas gingivalis*, *Tannerella forsythia*,* Treponema denticola*,* Prevotella intermedia*,* Campylobacter rectus*,* Filifactor alocis*, and *Fusobacterium nucleatum*) was determined by quantitative polymerase chain reaction (qPCR) as recently described [27]. The results were expressed by log_10_ counts/sample. Additionally, to increase interpretability, the abundance of the periopathogens was categorized as non-detectable, detectable < 6 log_10_ units/ sample, and detectable ≥ 6 log_10_ units/ sample.

### Intra-examiner calibration

To determine the intra-rater reliability PD and CAL measurements were performed in 4 quadrants of 2 patients. The causal and systematic errors were calculated using the interclass correlation coefficient (ICC; two-way mixed, absolute agreement) and Dahlberg’s formula. The intra-rater reliability for the measurements was good with an ICC of 0.8 for PD and CAL. The systematic method error was 0.06 mm for PD and 0.10 mm for CAL.

### Statistics

Statistical analysis was conducted using SPSS 29 (IBM, Armonk, NY, USA). Primary outcome was the reduction of PD (ΔPD) subtracting PD at T1 or T2 from PD at baseline, secondary outcomes were ΔCAL (subtracting CAL at T1 or T2 from baseline CAL), BOP, plaque, pocket closure, level of IL-1β and MMP-8 and periopathobionts. The required number of patients for sufficient statistical power of ≥ 80% for the primary outcome (difference of 1 mm ΔPD at T2) assuming a dropout rate of 20% was calculated by means of an a priori power analysis according to an already published study [28]. Mean (M) and standard deviation (SD), as well as median (MD) and interquartile range (IQR) were reported as descriptive statistics. Due to deviation from normal distribution according to visual assessment of histograms and Shapiro-Wilk tests, Friedman’s two-way analysis of variance by ranks was used for intragroup changes followed by post hoc pairwise comparison and Mann–Whitney U tests for intergroup comparison with Bonferroni correction. Nominal data was analyzed by Pearson Chi^2^ test. The level of statistical significance was set to *p* < 0.05.

## Results

### Baseline data

**Fig. 2:**
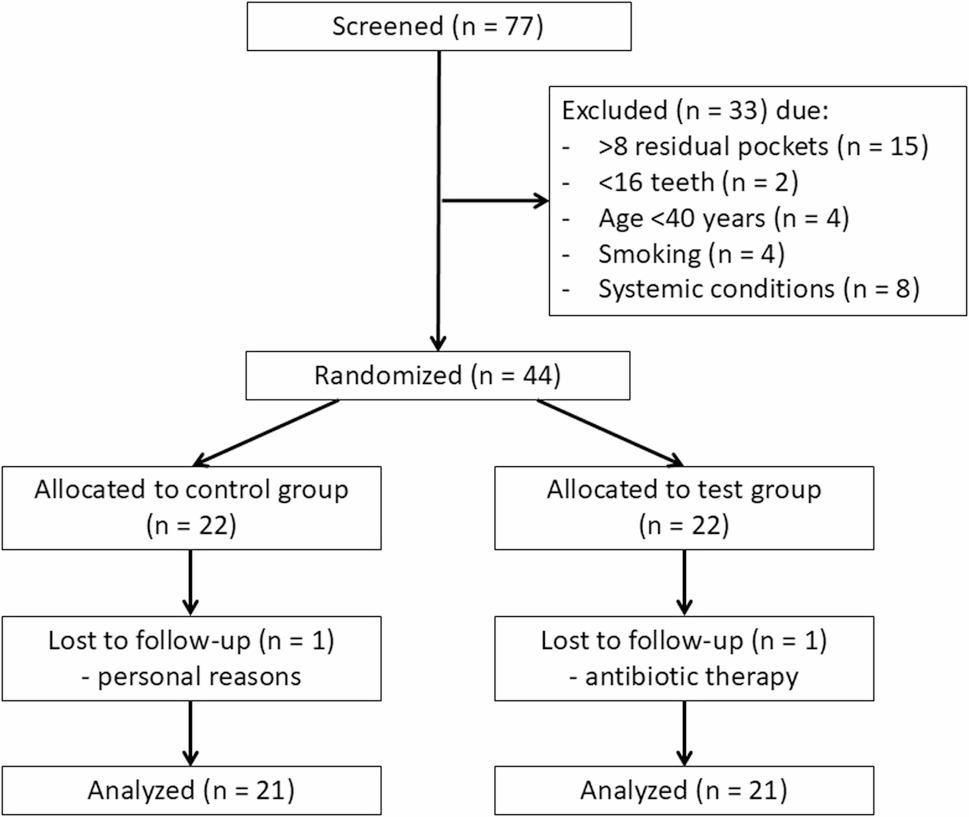
Flow chart according to the CONSORT criteria

During the recruitment period 77 patients were screened. Recruitment was stopped when 44 participants, predetermined by statistical power analysis, were randomly recruited, 22 for each treatment arm. Due to 1 loss in the follow-up per group, 21 subjects could be analyzed (Fig. 2). In both groups 71% were female. The mean age was 62.95 (± 7.46) vs. 67.23 (± 12.67) years, 25.48 (± 2.86) vs. 23.28 (± 4.1) mean number of teeth and 4.19 (± 1.36) vs. 4.29 (± 1.38) mean residual pockets in the control vs. test group. The proportion of multi-rooted teeth in the study sites was 78.6% (*n* = 33) in the control and 69.0% (*n* = 29) in the test group without a statistically significant difference between the groups (*p* = 0.321). Full mouth BOP and API were comparable (S2) in both groups as well as baseline measurements (Table 1).

**Table 1:** Descriptive and analytical outcomes of the study sites at different time points

			T0			T1			T2			ΔT0-T1			ΔT1-T2			ΔT0-T2			Frieman test	Post hoc tests		
Variable	Group	*n*	M	min	MD	M	min	MD	M	min	MD	M	min	MD	M	min	MD	M	min	MD	*p*-value	T0-T1	T1-T2	T0-T2
			SD	max	IQR	SD	max	IQR	SD	max	IQR	SD	max	IQR	SD	max	IQR	SD	max	IQR				
PD	control	21	**4.93**	4,00	**5.00**	**4.26**	2.00	**4.00**	**4.05**	2.00	**4,00**	**-0.67**	-2.00	**-1.00**	**-0.21**	-1.00	**0.00**	**-0.88**	-3.00	**-1.00**	< 0.001	0,001	0.826	< 0.001
			1.05	8.00	1.00	1.04	7.00	1.00	0.96	6.00	2.00	0.65	0.00	1.00	0.52	1.00	1.00	0.83	0.00	-1.25				
	test	21	**4.69**	4.00	5.00	**3.60**	2.00	**3.00**	**3.31**	2.00	**3.00**	**-1.10**	-3.00	**-1.00**	**-0.29**	-1.00	**0.00**	**-1.38**	-4.00	**-1.50**	< 0.001	< 0.001	0.518	< 0.001
			0.87	8.00	1.00	0.73	6.00	1.00	0.72	6.00	1.00	0,73	0.00	1.00	0.55	1.00	1.00	0.85	0.00	1.00				
*p*-value	intergroup		0.282			0.001			< 0.001			0.007			0.519			0.008						
CAL	control	21	**5.43**	4.00	**5.00**	**5.12**	3.00	**5.00**	**4.93**	3.00	**4.50**	**-0.31**	-2.00	**0,00**	**-0.19**	-1.00	**0.00**	**-0.50**	-2.00	**0,00**	< 0.001	0.243	0.826	0.014
			1.38	8.00	3.00	1.42	8.00	2.00	1.52	8.00	2.00	0.60	1,00	1.00	0.51	1.00	0.25	0.83	1.00	1.00				
	test	21	**5.14**	4.00	**5.00**	**4.48**	3.00	**4.00**	**4.07**	1.00	**4.00**	**-0.67**	-3.00	**-1.00**	**-0.40**	-2.00	**0.00**	**-1.07**	-4.00	**-1.00**	< 0.001	0.004	0.341	< 0.001
			1.39	10.00	2.00	1.45	9.00	1.00	1.40	8.00	2.00	0.87	2.00	1.00	0.77	1.00	1.00	1.20	2.00	2.00				
*p*-value	intergroup		0.299			0.020			0.015			0.017			0.194			0.017						

Differences in the median values (bold) between time-points were tested with nonparametric Friedman’s two-way analysis of variance by ranks for intragroup changes (rows) followed by post hoc pairwise comparisons and with Mann–Whitney U tests for intergroup comparison with Bonferroni correction (columns)

*CAL* clinical attachment level, *IQR* interquartile range, *M* mean, *MD* median, *n* numbers of analyzed patients, *PD* probing depth, *SD* standard deviation, *T0* baseline, *T1* 3 months after treatment, *T2* 6 months after treatment

### Clinical parameters

Subgingival re-instrumentation reduced PD in study sites from 4.93 mm (± 1.05; MD: 5.00 mm) to 4.26 mm (± 1.04; MD: 4.00 mm) at T1 and to 4.05 mm (± 0.96; MD: 4.00 mm) at T2 in the control group (Table 1). The PD in the test group was 4.69 mm (± 0.87; MD: 5.00 mm) at baseline, 3.60 mm (± 0.73; MD: 3.00 mm) at T1, and 3.31 mm (± 0.72; MD: 3.00 mm) at T2, respectively. The additional ΔPD in the test group was 0.43 mm (1.10, 95% CI range: 0.87–1.32 vs. 0.67, 95% CI range: 0.46–0.87; *p* = 0.007) at T1 and 0.50 mm (1.38, 95% CI range: 1.11–1.65 vs. 0.88, 95% CI range: 0.62–1.14; *p* = 0.008) at T2. The additional effect for ΔCAL at T2 was 0.57 (1.07, 95% CI range: 0.70–1.44 vs. 0.50, 95% CI range: 0.24–0.76; *p* = 0.017). The decrease in study sites BOP + at T2 was 19.0% in the control, and 78.6% in the test group (intergroup p-value < 0.001). Plaque at the study sites decreased in both groups and was significantly lower in the test group at T0 and T2 (Table 2). Residual pockets decreased from 100% in both groups by 38.1% (*n* = 16) in the control, and by 88.1% (*n* = 37) in the test group after 6 months (intergroup p-value < 0.001).

**Table 2:** Descriptive and analytical outcome of the study sites at different time points

			T0	T1	T2
Variable	Group	*n*			
BOP	control	21	40 (95.2%)	33 (78.6%)	32 (76.2%)
	test	21	38 (90.5%)	12 (28.6%)	5 (11.9%)
*p*-value	intergroup		0.397	< 0.001	< 0,001
Plaque	control	21	30 (71.4%)	13 (31.0%)	9 (21.4%)
	test	21	21 (50.0%)	11 (26.2%)	3 (7.1%)
*p*-value	intergroup		0.044	0.629	0,061
PD ≤ 4 mm BOP-	control	21	0 (0%)	12 (28.6%)	16 (38.1%)
	test	21	0 (0%)	34 (81.0%)	37 (88.1%)
*p*-value	intergroup		n.a.	< 0.001	< 0,001
PD ≥ 4 mm BOP+	control	21	42 (100%)	30 (71.4%)	26 (61.9%)
	test	21	42 (100%)	8 (19.0%)	5 (11.9%)
*p*-value	intergroup		n.a.	< 0.001	< 0,001
PD = 5 mm	control	21	21 (50.0%)	11 (26.2%)	11 (26.2%)
	test	21	22 (52.4%)	3 (7.1%)	1 (2.4%)
*p*-value	intergroup		0.827	0.019	0,002
PD ≥ 6 mm	control	21	7 (16.7%)	5 (11.9%)	3 (7.1%)
	test	21	4 (9.5%)	1 (2.4%)	1 (2.4%)
*p*-value	intergroup		0.332	0.090	0.306

Results were calculated using crosstab and intergroup comparison were performed by Pearson Chi^2^ test

*T0* baseline, *T1* 3 months after treatment, *T2* 6 months after treatment, *BOP* bleeding on probing

### Biomarkers and microbiology

**Fig. 3:**
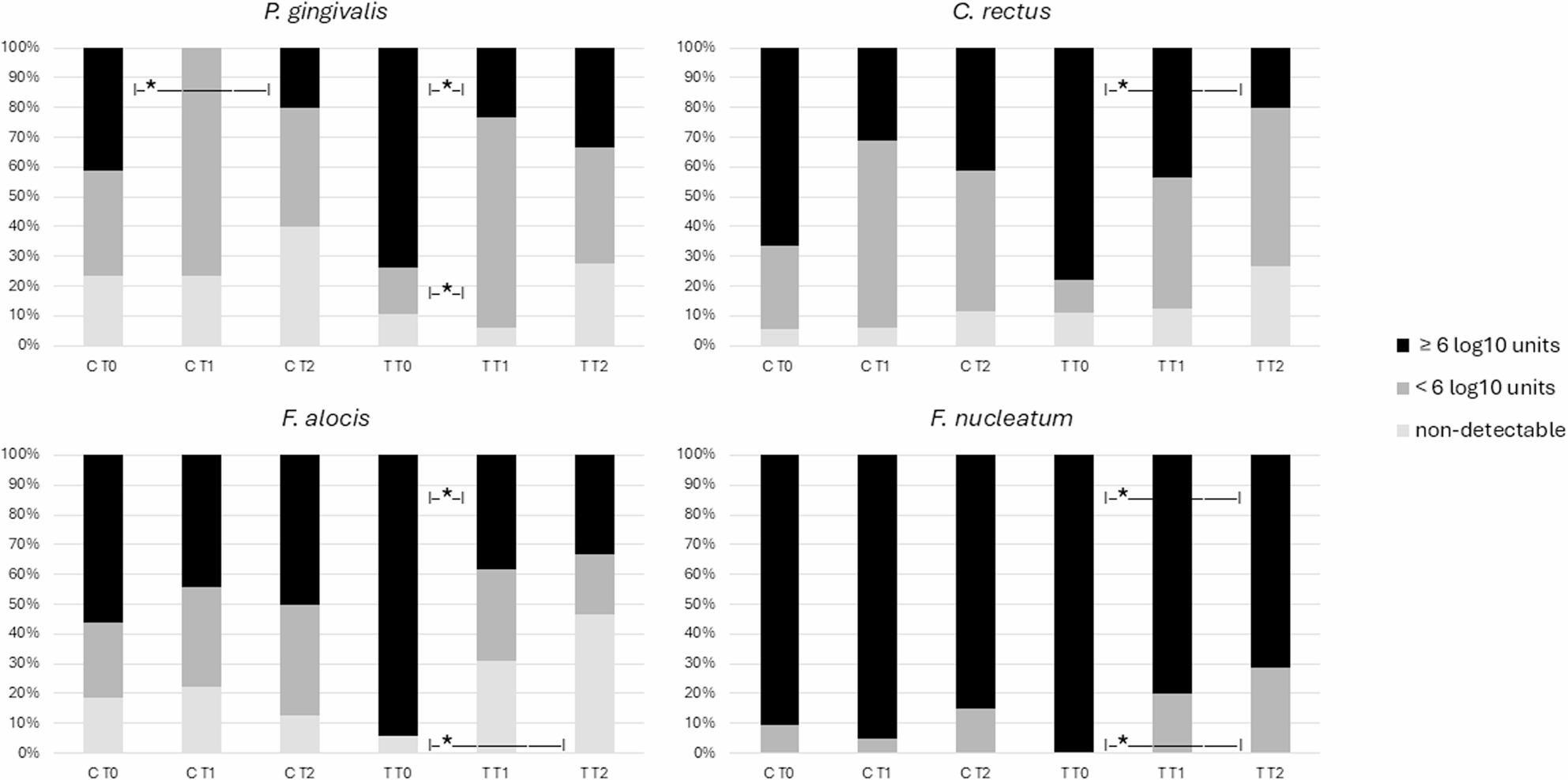
Categories (non-detectable, < 6 log10 units, ≥ 6 log10 units) of abundance for selected periopathobionts at the different time points (T0-T2), C: control group, T: test group, T0: baseline, T1: 3 months after treatment, T2: 6 months after treatment, * indicates statistically significant differences (*p* < 0.05)

For IL-1β and MMP-8, the reduction neither reached statistical significance at any time-point, nor was the level different between the groups (S3). The abundance of the tested periopathobionts is presented in supplementary data (S4). A trend to a reduced abundance could be observed for every studied periopathobiont over time. The decreases of *P. gingivalis*,* T. denticola*,* C. rectus*,* F. alocis*, and *F. nucleatum* in the test group were statistically significant. Figure 3 shows the categories of abundance of selected periopathobionts.

## Discussion

The aim of the study was to investigate the additional effects of AA-NaOCl and xHA combined with SRI on clinical periodontal parameters, immunological biomarkers and the subgingival microbiota after 3 and 6 months in a RCT design. Both groups showed an improved outcome compared to baseline. No adverse effects were observed. The reduction of PD of 0.88 mm 6 months after SRI is in concordance with the reported range of 0.7 to 1.9 mm [29]. It must be noted that the results in the latter review were achieved using a rubber cup or brushes for polishing, in contrast to the present study, where air polishing was performed. But polishing with conventional methods and air polishing were found to be comparable [30]. A very recent study using the same treatment protocol for both test and control group, demonstrated a higher reduction of ΔPD after 3 months (1.34 mm vs. 0.67 mm in the present study). A possible explanation could be found in the inclusion criteria. The patient cohort in the present study exhibited a better controlled periodontal status indicated by an interproximal plaque index (API) ≤ 35% and a maximum of 8 residual pockets compared to API ranging from 20 to 60% and a mean number of 30 residual pockets per patient [24]. The application of AA-NaOCl and xHA resulted in an additional ΔPD of 0.50 mm and ΔCAL of 0.57 mm after 6 months compared to SRI alone. The included participants were initially diagnosed with periodontitis stage III or IV and therefore a 3-months SPC interval was indicated [31]. Repeated instrumentation will not interfere with the healing process as the major changes in PD occur in the first 1–2 months after SRI [32]. Thus, at T1 (3 months after baseline), pockets with a persistent need for treatment were re-instrumented according to the allocated protocol.

In a RCT investigating the effect during SRI, the adjunctive application of AA-NaOCl demonstrated a trend to an additional ΔPD of 0.21 mm compared to saline and 0.31 mm compared to a 1% chlorhexidine (CHX) gel (*p* = 0.069), and additional ΔCAL of 0.24 mm and ΔCAL of 0.46 mm, respectively [33]. The results in the present research are more pronounced, which may be due to the additional application of xHA.

The subgingival application of HA after SRI and supragingival on a daily basis resulted in an additional ΔPD of 0.2 mm compared with SRI at 3 months [34]. The additional reduction of 0.36 mm compared to control found in our study may be the cumulative effect of both adjuncts.

Other locally delivered antimicrobial adjuncts to SRI may be sustained-release CHX and tetracycline fibres. The additional ΔPD found in a systematic review was 0.6 to 0.7 mm [35]. The additional ΔPD after the flapless application of enamel matrix derivatives was 0.79 ± 1.3 mm (*p* < 0.0001) after 6 months [36]. This is superior to the additional effect found in our study, whereas the clinical application of enamel matrix derivatives is clinically more challenging, and therefore the protocol presented here may be easier to be implemented.

For initial therapy, an additional ΔPD of 1.1 mm and a ΔCAL of 0.7 mm was shown for AA-NaOCl and xHA in moderate pockets (4–6 mm PD) and ΔPD of 2.1 mm and a ΔCAL of 2.0 mm in deep pockets (7 mm PD) after 6 months, respectively [23]. In this study pockets were treated for the first time during anti-infective therapy. Therefore, a greater impact of AA-NaOCl and xHA on the resolution of the inflammation could be demonstrated. The sites included in the present study have been already treated before and did not respond satisfactory to the initial treatment. Lower responsiveness of these patients may explain the lower results of the present study during SRI in comparison to SI.

In a recent RCT, AA-NaOCl and xHA were adjunctively applied during SRI [24]. The test group showed an additional ΔPD of 0.10 mm after 3 months and 0.25 mm after 9 months, and a ΔCAL of 0.7 mm and 0.61 mm compared to the control group, but no difference in BOP. These results were lower than ours. The median PD at baseline was 4 mm, compared to 5 mm in our study. Adjunctive AA-NaOCl and xHA may have a higher impact on the PD reduction in deeper pockets as we demonstrated in our subgroup analysis. This may explain the greater reduction in the present study.

Levels of IL-1β and MMP-8 levels in the GCF are elevated in pockets compared to inflammation-free sites and after step 2 treatment these elevated levels decrease [37]. In this study the levels did not change significantly. The data on the alteration of IL-1β level is ambiguous. A short-term decrease after instrumentation, but also an increase as an expression of tissue remodeling is reported. Monitored during SPC, IL-1β levels in the GCF remain stable and are probably more related to general inflammation of the patient than to local reaction [38].

A statistically significant reduction of *P. gingivalis*,* T. denticola*,* C. rectus*,* F. alocis*, and *F. nucleatum* was found in the test group only. A reduction of periopathobionts is mainly seen after the initial therapy (step 2) [39]. For SPC, stability in the periodontal microbiota, i.e. no significant changes in any direction, was also confirmed in literature [40–42]. After SRI in SPC patients, the levels of periopathobionts decreased, but reached pre-treatment levels 3 months later [43]. Little is known about the microbiological effects of HA in clinical application. One group found a delayed recolonization of the pockets with *C. rectus* and *P. gingivalis* [44], whereas in another study, HA exhibited no effect on *P. gingivalis* [45]. No change in the subgingival microbiota could be observed after AA-NaOCl combined with SRI [33]. The application of AA-NaOCl and xHA during initial instrumentation resulted in a reduced abundance of *P. gingivalis*, *T. denticola*, and *P. intermedia* after 6 months, whereas the counts in the control group remained stable [46]. The presence of *P. gingivalis* might be a negative predictive factor for treatment response [47, 48] and might increase the risk for progressive alveolar bone loss with an odds ratio of 31.9 [49]. Reducing the counts of *P. gingivalis* with the adjunctive protocol may therefore facilitate periodontal healing.

There are some limitations to consider. Due to the taste and smell of NaOCl in the tested gel formulation, blinding for the treatment was impossible, but the investigator for the sample analysis was blinded. The microbiological samples were collected using paper points being a standard method [36, 47, 50, 51]. Studies comparing paper point and curette demonstrated a good agreement between both methods [52, 53]. Compared to multi-center studies less participants could be included. On the other hand, all measurements were taken by only one experienced investigator, and a sample size calculation was performed a priori. The limited number of participants is a limitation, especially for assessing confounding variables for treatment success, e.g. initial PD, number of roots, furcation involvement, osseous defect angulation among others [54]. Patients with a need for non-surgical reinstrumentation were included but it was not differentiated in what course of retreatment they have been within the systematic therapy. In this study the two pockets with the highest PD were treated with AA-NaOCl and xHA in the treatment group and the two highest PD were included in the control group. This contrasts with other investigations, where all remaining pockets got adjunctive treatment [24] and results in a lower number of investigated sites. The rationale behind this decision was to ensure an equal number of sites were included for each patient. According to the inclusion criteria, a minimum of two persisting pockets was necessary, bearing in mind that this study is not focused on the initial periodontal treatment. This is in accordance with other published studies [55–59]. The presented protocol did not show any adverse effects. The assistant must suction well during the application of NaOCl and the respective tooth must be isolated with cotton rolls. This increases the patient’s comfort and perception of treatment. In general, adjunctive treatments seem to have more impact and benefits in higher PDs [23, 24, 29, 60]. Regarding additional expenditure in terms of costs for the material and a prolonged treatment time it is reasonable to apply the additional measures in the pockets that gain the most of it. Therefore, the focus in this study was on the two sites with highest measured PD to reflect a clinical realistic setting and to avoid overtreatment. Further studies with a higher number of patients and a longer follow-up are needed to confirm these results.

## Conclusion

The adjunctive application of AA-NaOCl and xHA in combination with SRI resulted in an additional benefit for ΔPD and ΔCAL and a higher rate of pocket closure compared to mere SRI. The PD reduction was more pronounced in sites with higher initial PD. Furthermore, a decrease in the abundance of 5 of the 8 analyzed periopathobionts could be observed in the test group. Therefore, the application may be a reasonable method to achieve a better clinical outcome in sites with a need for SRI but should be predominantly used in pockets with higher probing depths. 

## Supplementary Information

Supplementary Material 1.

## Data Availability

All data supporting the findings of this study are available within the paper and its Supplementary Information. The datasets used and/or analyzed during the current study are available from the corresponding author on reasonable request.
