# Supplementary material for: Effect of sodium hypochlorite and hyaluronic acid in subgingival re-instrumentation – a randomized clinical trial
Source: BMC Oral Health. 2026 May 30;26:1022. doi: 10.1186/s12903-026-08694-9 (PMC13262014; doi:10.1186/s12903-026-08694-9)
Supplement: Supplementary file 1 — Supplementary Material 1. [file 12903_2026_8694_MOESM1_ESM.docx]

# Effect of Sodium Hypochlorite and Hyaluronic Acid in Subgingival Re-Instrumentation – a Randomized Clinical Trial

## Supplementary materials

S1: Timeline for the included study participants


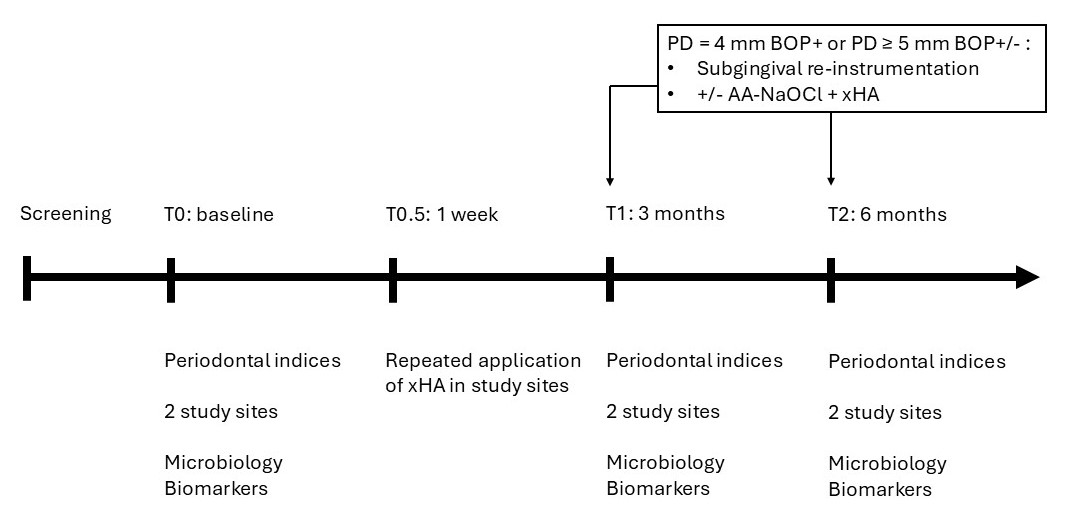


S2: Baseline data and statistics

| Variable (±SD) | Control (n = 21) | Test (n = 21) | p-value |
| --- | --- | --- | --- |
| Age (years) | 62.95 (±7.46) | 67.24 (±12.67) | 0.082 |
| Gender male/ female (n) | 6/ 15 | 6/ 15 | n.a. |
| Smoking (n) | 1 | 1 | n.a. |
| Teeth (n) | 25.48 (±2.86) | 23.38 (±4.10) | 0.063 |
| Mean PD (mm) | 2.50 (±0.23) | 2.55 (±0.32) | 0.760 |
| Total residual pockets (n) | 88 | 90 | n.a. |
| Mean residual pockets (n) | 4.19 (±1.36) | 4.29 (±1.38) | 0.835 |
| Mean CAL (mm) | 3.10 (±0.59) | 3.01 (±0.44) | 0.930 |
| BOP (%) | 21.65 (±8.08) | 19.21 (±6.83) | 0.378 |
| API (%) | 26.05 (±6.34) | 23.51 (±7.94) | 0.278 |
| SBI (%) | 4.46 (±4.02) | 4.94 (±3.89) | 0.646 |

*Mann–Whitney U test was applied for intergroup comparison, API interproximal plaque index, BOP bleeding on probing, CAL clinical attachment level,, n.a. not applicable, PD probing depth, SBI sulcus bleeding index, SD standard deviation*

S3: Levels of IL-1β and MMP-8 in GCF in pg/ sample


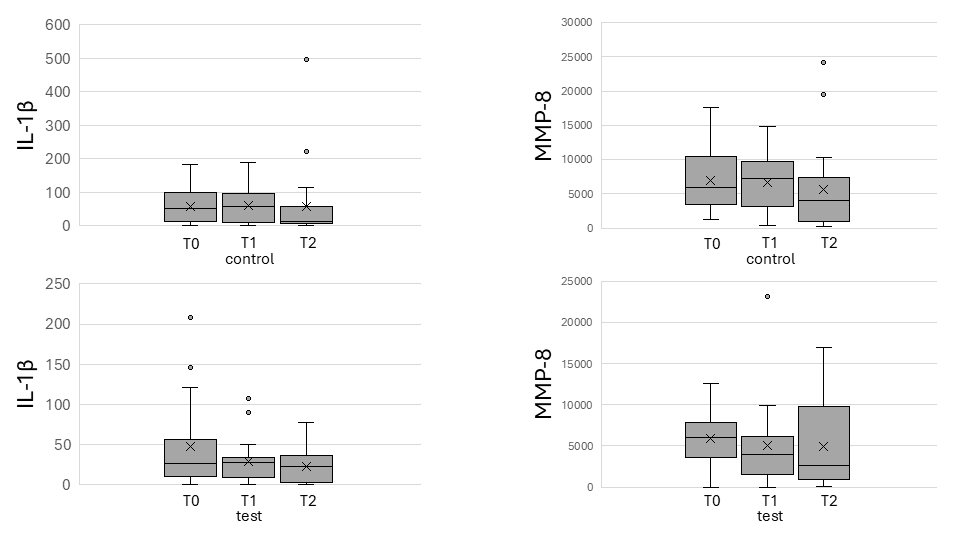


*Global changes were tested with nonparametric Friedman’s two-way analysis of variance by ranks for intragroup changes (rows) followed by post hoc pairwise comparisons and with Mann–Whitney U tests for intergroup comparison (columns), IL-1β Interleukin-1beta, MMP-8 matrix metalloproteinase-8, T0 baseline, T1 3 months after treatment, T2 6 months after treatment*

S4: Descriptive and analytical outcome of the abundance of the tested periopathobionts by log_10_ counts/sample during the different time periods.

|  | | **T0** | | | **T1** | | | **T2** | | | **Friedman test** | **Post hoc tests** | | |
| --- | --- | --- | --- | --- | --- | --- | --- | --- | --- | --- | --- | --- | --- | --- |
| log_10_ counts/site | Group | M | min | MD | M | min | MD | M | min | MD | p-value | T0-T1 | T1-T2 | T0-T2 |
|  |  | SD | max | IQR | SD | max | IQR | SD | max | IQR |  | | | |
| **Aa** | Control | **0.28** | 0.00 | 0.00 | **0.76** | 0.00 | 0.00 | **1.22** | 0.00 | 0.00 | 0.411 | n.a. | n.a. | n.a. |
|  |  | 1.29 | 8,00 | 0.00 | 1.90 | 5.47 | 0.00 | 2.28 | 6.28 | 0.00 |  | | | |
|  | Test | **0.82** | 0.00 | 0.00 | **0.25** | 0.00 | 0.00 | **0.50** | 0.00 | 0.00 | 0.146 | n.a. | n.a. | n.a. |
|  |  | 2.02 | 6.23 | 0.00 | 0,79 | 4.99 | 0.00 | 1.53 | 5.31 | 0.00 |  | | | |
| p-value | Intergroup | 0,311 | | | 0.311 | | | 0.388 | | |  |  |  |  |
| **Pg** | Control | **3.63** | 0.00 | 4.86 | **3.02** | 0.00 | 3.91 | **2.41** | 0.00 | 0.00 | 0.368 | n.a. | n.a. | n.a. |
|  |  | 3.04 | 7.17 | 6.60 | 2.53 | 5.84 | 5.66 | 2.90 | 6.76 | 5.46 |  | | | |
|  | Test | **5.33** | 0.00 | 6.30 | **4.29** | 0.00 | 5.32 | **3.47** | 0.00 | 4.28 | 0.001 | 0.043 | 1.000 | 0.004 |
|  |  | 2.45 | 7.56 | 2.25 | 2.38 | 6.77 | 2.62 | 2.78 | 6.49 | 6.05 |  | | | |
| p-value | Intergroup | 0.112 | | | 0.86 | | | 0.318 | | |  |  |  |  |
| **Tf** | Control | **5.33** | 0.00 | 5.86 | **4.34** | 0.00 | 5.90 | **4.44** | 0.00 | 5.88 | 0.791 | n.a. | n.a. | n.a. |
|  |  | 1.19 | 7.02 | 1.19 | 2.83 | 6.78 | 6.28 | 2.94 | 7.33 | 6.57 |  | | | |
|  | Test | **6.10** | 0.00 | 6.43 | **5.22** | 0.00 | 6.10 | **4.31** | 0.00 | 5.76 | 0.002 | 0.017 | 1.00 | 0.005 |
|  |  | 1.49 | 7.19 | 0.77 | 2.27 | 6.65 | 0.67 | 2.94 | 6.79 | 6.52 |  | | | |
| p-value | Intergroup | 0.039 | | | 0.319 | | | 0.601 | | |  | | | |
| **Td** | Control | **3.95** | 0.00 | 5.53 | **3.28** | 0.00 | 5.01 | **2.53** | 0.00 | 0.00 | 0.185 | n.a. | n.a. | n.a. |
|  |  | 2.66 | 6.31 | 6.07 | 2.94 | 6.33 | 6.04 | 3.00 | 6.62 | 5.82 |  | | | |
|  | Test | **4.44** | 0.00 | 5.75 | **3.72** | 0.00 | 5.50 | **3.44** | 0.00 | 5.05 | 0.084 | n.a. | n.a. | n.a. |
|  |  | 2.69 | 6.83 | 5.20 | 2.85 | 6.34 | 6.09 | 2.93 | 7.01 | 5.91 |  | | | |
| p-value | Intergroup | 0.303 | | | 0.619 | | | 0.581 | | |  |  |  |  |
| **Pi** | Control | **3.31** | 0.00 | 5.39 | **2.58** | 0.00 | 0.00 | **1.92** | 0.00 | 0.00 | 0.192 | n.a. | n.a. | n.a. |
|  |  | 3.11 | 7.31 | 6.05 | 2.96 | 6.95 | 5.69 | 2.74 | 6.24 | 5.29 |  | | | |
|  | Test | **2.88** | 0.00 | 0.00 | **2.38** | 0.00 | 0.00 | **2.11** | 0.00 | 0.00 | 0.050 | 1.000 | 0.291 | 1.000 |
|  |  | 3.30 | 7.35 | 6.17 | 3.02 | 7.33 | 5.78 | 2.98 | 7.15 | 5.77 |  | | | |
| p-value | Intergroup | 0.860 | | | 0.953 | | | 0.926 | | |  |  |  |  |
| **Cr** | Control | **5.04** | 0.00 | 6.26 | **4.36** | 0.00 | 5.50 | **4.39** | 0.00 | 5.76 | 0.265 | n.a. | n.a. | n.a. |
|  |  | 2.71 | 7.57 | 2.37 | 2.68 | 7.12 | 5.04 | 2.69 | 6.72 | 5.43 |  | | | |
|  | Test | **5.19** | 0.00 | 6.45 | **3.91** | 0.00 | 4.94 | **3.00** | 0.00 | 4.22 | <0.001 | 0.027 | 1.000 | 0.003 |
|  |  | 3.14 | 7.82 | 6.08 | 2.74 | 6.86 | 6.21 | 2.85 | 6.86 | 5.75 |  | | | |
| p-value | Intergroup | 0.240 | | | 0.792 | | | 0.61 | | |  |  |  |  |
| **Fa** | Control | **4.12** | 0.00 | 5.85 | **4.22** | 0.00 | 5.54 | **4.21** | 0.00 | 5.51 | 0.985 | n.a. | n.a. | n.a. |
|  |  | 3.16 | 7.32 | 6.72 | 2.88 | 7.19 | 6.22 | 2.90 | 6.98 | 6.43 |  | | | |
|  | Test | **5.22** | 0.00 | 6.65 | **2.70** | 0.00 | 0.00 | **2.45** | 0.00 | 0.00 | <0.001 | 0.006 | 1.000 | 0.006 |
|  |  | 3.16 | 8.42 | 5.85 | 3.09 | 6.78 | 6.03 | 3.11 | 6.92 | 6.04 |  | | | |
| p-value | Intergroup | 0.061 | | | 0.270 | | | 0.079 | | |  |  |  |  |
| **Fn** | Control | **7.18** | 5.41 | 7.20 | **7.03** | 4.74 | 6.97 | **6.71** | 4.41 | 6.89 | 0.157 | n.a. | n.a. | n.a. |
|  |  | 0.74 | 8.29 | 0.70 | 0.90 | 8.86 | 1.27 | 0.78 | 7.57 | 0.64 |  | | | |
|  | Test | **7.38** | 6.01 | 7.32 | **6.93** | 4.48 | 7.12 | **6.62** | 5.39 | 6.75 | 0.001 | 0.464 | 0.053 | <0.001 |
|  |  | 0.61 | 8.54 | 0.76 | 1.10 | 8.82 | 1.54 | 0.60 | 7.39 | 1.09 |  | | | |
| p-value | Intergroup | 0.399 | | | 0.735 | | | 0.251 | | |  |  |  |  |

*Global changes were tested with nonparametric Friedman’s two-way analysis of variance by ranks for intragroup changes (rows) followed by post hoc pairwise comparisons and with Mann–Whitney U tests for intergroup comparison (columns), IQR interquartile range, M mean, MD median, n.a. not applicable, T0 baseline, T1 3 months after treatment, T2 6 months after treatment*
